# Effects of a Novel NiTi Thermomechanical Treatment on the Geometric Features of the Prepared Root Canal System

**DOI:** 10.3390/ma13235546

**Published:** 2020-12-04

**Authors:** Abdulwahed Alghamdi, Loai Alsofi, Khaled Balto

**Affiliations:** Department of Endodontics, Faculty of Dentistry, King Abdulaziz University, Jeddah 21589, Saudi Arabia; dr.a.wahed1@gmail.com (A.A.); kbalto@kau.edu.sa (K.B.)

**Keywords:** root canal preparation, dental alloys, martensite, austenite, X-ray micro-CT

## Abstract

**Objectives:** This study aimed to compare the ex vivo performance of two rotary nickel–titanium (NiTi) systems with similar designs but manufactured from martensitic and austenitic alloys, the One Curve (OC) and One Shape (OS) rotary endodontic files, respectively. Methods: Forty separate mesial canals of 20 extracted mandibular molars were scanned using micro-computed tomography (CT), which were divided into 2 groups and instrumented with OC and OS, respectively. Post-instrumentation micro-CT scans were evaluated using validated computer algorithms to compare changes in canal thickness, surface area, structure model index (SMI) scores, volume of removed dentin, percentage of untreated canal surface, percentage of curvature straightening, and the amount of canal transportation. Results: Both files led to significant changes in the basic root canal geometry, with no preparation errors and no statistically significant differences. However, OC treatment resulted in significantly less curvature straightening (17.30%; 10.77%) (independent samples *t* test, *p* < 0.05) and less apical transportation (55.11 µm; 33.15 µm) (Mann–Whitney *U,* test *p* < 0.05) compared to OS treatment. Transportation values in the middle and coronal thirds were statistically similar (independent sample *t*-test, *p* > 0.05). OC treatment produced significantly less straightening and less apical transportation than OS.

## 1. Introduction

The number of root canal treatments has increased considerably over the years due to advances in restorative modalities and increased awareness regarding preservation of natural teeth [1]. During root canal treatment, the instrumentation enables irrigation, disinfection, and obturation. The era of rotary endodontic files was marked by the introduction of nickel–titanium (NiTi) rotary files into endodontics in the early nineties. Before this, stainless steel (SST) was the only material used to manufacture endodontic instruments. NiTi alloy was first developed in 1963 by Buehler et al. In their work, 56% of the weight of the developed alloy was nickel and the remaining 44% was titanium. It was shown that this composition of metals in the alloy gives it the property of super-elasticity or shape memory. The alloy was named nitinol after the Naval Ordinance Laboratory, the laboratory where the development of the alloy took place [2,3]. Soon after, NiTi gained a lot of popularity in orthodontics after it was introduced clinically in 1975. The physical properties of NiTi are more favorable for clinical use than SST. NiTi is more flexible than SST due to its lower modulus of elasticity, resulting in lower values in bending tests [4]. Since their introduction, NiTi endodontic files have undergone various design modifications by manufacturers to simplify their use and enhance their performance. Recent advances in metallurgical engineering have led to the development of a new generation of NiTi rotary files that are manufactured by different heat treatments, rendering various products such as M-wire, R-phase, controlled memory (CM), Max-wire, T-wire, and C-wire instruments [5,6,7,8]. These heat-treated files exhibit different phase transformation behavior, which improves their flexibility, fatigue resistance, and torsional resistance in vitro [9,10,11,12,13,14]. The currently marketed heat-treated files have a wide range of designs, cross-sections, sizes, and motion kinematics. With these fundamental design differences, previous studies that compared the shaping abilities of martensitic and conventional austenitic NiTi endodontic files might have contained confounding bias in their results [15,16,17].

Coltene Micro-Mega (Besancon, France) introduced the One Curve (OC) file system, which is a martensitic NiTi file. The file has a variable cross-section and pitch to reduce the instrument threading and binding in the canal walls. Interestingly, the One Shape (OS) (Coltene Micro-Mega, Besancon, France) instrument is a conventional austenitic NiTi that exhibits the same design features (for example, cross-section, size, taper, and motion kinematics) as the OC instrument [18]. Therefore, the aim of this research is to compare the shaping abilities of the novel thermomechanical treatment with the OC rotary file to the conventional NiTi OS rotary file in the mesial canals of mandibular molars. The null hypothesis was that there would be no significant difference in the shaping abilities of the OC or OS rotary file systems in the mesial canals of mandibular molars.

## 2. Materials and Methods

### 2.1. Sample Selection and Randomization

The institution’s research ethics committee approved this project before its commencement (number: 016-01-18). The first and second mandibular molars that were extracted because of severe periodontal diseases were collected. The teeth were examined under magnification, and radiographs were obtained to select teeth that met the following inclusion criteria: mesial roots of mandibular first and second molars with two distinct canals, and roots with fully mature apices and canal curvatures between 20 and 45 degrees [19]. Teeth with extensive carious lesions or crown fracture were excluded. Unusual anatomies such as extensive calcifications, pulp stones, resorptive defects, or extreme curvature were eliminated from the sample. The selected (*n* = 20) teeth were kept in 0.1% NaOCl at 5 °C, and their mesial canals were randomly allocated to either the OC group or OS group (*n* = 20). A web-based software (Random.Org) was used to ensure random and equal allocation for mesiobuccal and mesiolingual canals to both groups. Each group acted as its own control group with the pre- and post-instrumentation measurements.

### 2.2. Root Canal Instrumentation

The teeth were mounted on micro-computed tomography (CT) sample holders and the first micro-CT scans were obtained. This mounting on the sample holders ensured the scanning of samples in the same position before and after instrumentation [20]. The access cavity was prepared using a tapered round-end diamond bur. A #10 K-file was used to negotiate the canals and to verify their patency radiographically. Instrumentation protocols recommended by the manufacturers were followed for both groups. The One Flare (COLTENE Micro-Mega, Besancon, France) orifice opener was used to obtain straight-line access to the coronal thirds of the canals. The working length was measured using number 10 K-type file up to 0.5–1 mm from the radiographic apex. The #15 K-files and One G path files (COLTENE Micro-Mega, Besancon, France) were used to establish a glide path. Instrumentation for teeth in both groups was performed using the Elements Motor instrument (SybronEndo, Orange, CA, USA) at 350 rpm and 1.2 N.cm torque with the crown-down technique and with picking motion advancing the instrument no more than 3 mm at a time. At the same time, the canal was lubricated with SlickGel ES (SybronEndo, Orange, CA, USA) along with copious 5.25% NaOCl irrigation to flush the dentinal debris. Recapitulation was performed with number 10 K-type files after each instrumentation cycle to avoid canal blockage. This procedure was repeated until the pre-measured working length was engaged. After instrumentation, the canals were irrigated with 2 mL of 5.25% NaOCl for 30 s using a side-vented 30-gauge needle (Max-I-Probe, Dentsply Maillefer, Tulsa, OK, USA). Finally, the canals were soaked in 1 mL 17% EDTA for 1 min before the final 1 mL rinse with distilled water. All shaping procedures were completed by a single operator who was blinded to the pre-acquired micro-CT scans. The biomechanical preparation was completed under magnification and illumination using a dental operating microscope (OPMI PICO, Carl Zeiss, Oberkochen, Germany).

### 2.3. Micro-CT

**Figure 1:**
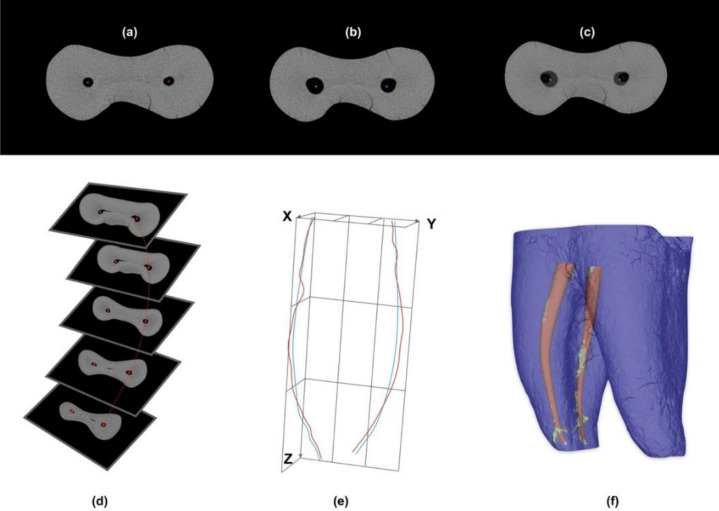
(**a**) Center of mass before instrumentation. (**b**) Center of mass after instrumentation. (**c**) Superimposition of the pre- and post-instrumentation scans to determine transportation. (**d**) Canal curvature calculated by using the mathematical formula of the second derivative of the connected centers of mass in the z-axis. (**e**) Curvature straightening expressed as the percentage of change in canal curvature after instrumentation. (**f**) Treated and untreated canal surfaces were evaluated by superimposing the pre- and post-instrumentation canal surface areas. The static voxels after superimposition resemble the untreated canal surfaces.

Pre- and post-instrumentation scans were obtained for each sample using the micro-CT 100 apparatus (Scanco Medical AG, Brüttisellen, Switzerland). The scanning settings were as follows: 90 kVp, 88 μA, and an integration time of 500 ms. The obtained data were reconstructed to an isotropic resolution of 14 μm using a filtered back-projection algorithm. The difference in contrast between the lumen of the empty canal and dentin allowed the segmentation of each micro-CT slice in order to calculate the canal surface area and volume. Accordingly, the structure model index (SMI) was used to categorize the volumetric structure of the canal as either being platter-like or rod-like. Canal thickness was calculated by averaging the diameter of the largest sphere that fit the volume structure. Canal transportation was assessed by defining the centers of mass of the canals in all axial slices pre-instrumentation, then comparing them with centers of mass post-instrumentation following superimposition. Canal transportation was evaluated in coronal, middle, and apical thirds. Canal curvature values were calculated using the mathematical formula of the second derivative of the connected centers of mass in the z-axis, and straightening was then expressed by the percentage of change in canal curvature due to instrumentation [20]. The treated and untreated canal surfaces were evaluated by superimposing the “before” and “after” canal surface areas. The static voxels after superimposition resembled the untreated canal surfaces [21]. These parameters were calculated using validated computer algorithms [22] (IPL V5.42 Scanco Medical AG) (Figure 1).

### 2.4. Statistical Analysis

The Shapiro–Wilk test was used to test the normality of the data. Independent sample *t-*tests for normally distributed data and Mann–Whitney U test for non-normally distributed data were used to compare the differences between the OC and OS groups for the measured parameters after instrumentation. All statistical analyses were performed using the IBM SPSS Statistics 22.0 (SPSS Inc., Chicago, IL, USA) at 0.05 level of significance.

## 3. Results

**Figure 2:**
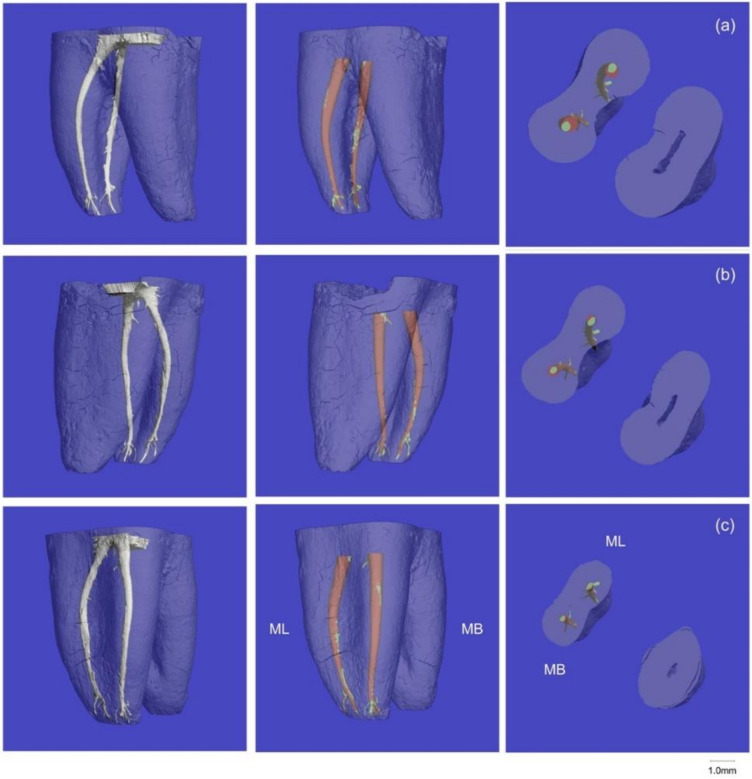
A representative sample of prepared root canals. The left column shows unprepared canals. The middle column shows the treated (red) and untreated (green) canal surfaces. The right column shows the canal cross-sections of the canals: (**a**) coronal, (**b**) middle, and (**c**) apical thirds. The mesiolingual (ML) canal was instrumented with One Curve (OC), and the mesiobuccal (MB) canal was instrumented with One Shape (OS).

Qualitative analysis of the three-dimensional micro-CT renderings showed no procedural errors (instrument separation, root perforations, ledges, zipping, or canal blockage) after instrumentation with both files. A representative example of root canals instrumented with both OS and OC is shown in Figure 2.

The independent sample *t-*tests showed no significant differences between groups (*p* > 0.05) for any pre-instrumentation parameters (canal thickness, surface area, volume, or SMI score), confirming the randomness of allocation and the homogeneity between the tested groups. Increases in the values of tested parameters after instrumentation with both files were observed. For example, the measured increases were 0.24 ± 0.09 mm, 2.61 ± 1.98 mm^2^, and 0.80 ± 0.58 for the canal thickness, surface area, and SMI score, respectively, after instrumentation with OS. The corresponding measured increases were 0.22 ± 0.09 mm, 1.87 ± 1.65 mm^2^, and 0.68 ± 0.58, respectively, after instrumentation with OC. Aside from the increases in canal surface area, SMI score, and canal thickness, no significant differences were observed between teeth in the OS and OC groups (*p* > 0.05) (Table 1).

The mean volume of removed dentin in teeth in the OC group was 1.02 ± 0.52 mm^3^, while that in teeth in the OS group was 1.37 ± 0.72 mm^3^, with no significant difference between the two groups (*p* = 0.093). The percentage of untreated canal surface in the teeth was 52.36 ± 17.83 % in the OC group, and that in the OS group was 54.52 ± 16.09 %. The independent sample *t-*test showed no significant difference between the two groups (*p* = 0.691).

Teeth in the OS group showed significantly higher values of 17.30 ± 11.86% for the straightening percentage than teeth in the OC group (10.77 ± 6.81%). Canal transportation was assessed in the coronal, middle, and apical thirds of each root (Table 1). Teeth in the OS group showed higher transportation along the entire root than teeth in the OC group. However, this difference was significant only in the apical third.

## 4. Discussion

This study investigated the ex vivo performance of OC and OS files. The shaping ability of endodontic instruments can be affected by several factors, including motion kinematics (continuous rotation, reciprocation, adaptive rotation, or oscillation), instrument design (size, taper, cross-section, and number of flutes), and number of instruments used [15,23,24,25,26,27]. Here, the analyzed instruments had the same design, size, taper, cross-section, and number of flutes. Therefore, all variables that could have affected the shaping ability of tested files were standardized [28]. Hence, the only factor that would have influenced the results was the metallurgical properties of these files. OS is an austenitic superelastic NiTi rotary file. Conversely, OC is martensitic in nature and manufactured by a thermomechanical treatment of NiTi.

The shaping abilities of both files were tested in an ex vivo model on extracted molars and were assessed using micro-CT [29,30], which is a non-destructive and precise method [31,32]. Usually, mesial roots of mandibular molars tend to have two distinct canals with moderate curvature [33]. Hence, the mesial roots of mandibular molars are considered ideal for testing the shaping abilities of different types of files [33]. This study showed comparable results for both instruments with no significant differences between them in canal thickness, surface area, SMI score, and volume of removed dentin. The similar design features of both files could explain these results. One goal of mechanical shaping of root canals is the removal of the inner infected dentin to facilitate delivery of irrigation and to mechanically disrupt the microbial biofilm [34]. The ability of endodontic files to come in contact with all surfaces of the root canals has been questioned [35]. Therefore, here the untreated canal walls were quantified by superimposing the pre- and post-instrumentation canal volumes to determine the percentages of static voxels to the total surface voxels. No significant difference was observed between canals prepared with either instrument—approximately 52–55% of untreated canal surface was observed after instrumentation with both instruments, as previously reported [20,35,36]. Canals instrumented with OS showed significantly more straightening of canal curvature and more transportation in the apical thirds compared to canals instrumented with OC. These results can be explained by the martensitic arrangement of the NiTi crystals in OC. Martensitic instruments have controlled memory with enhanced flexibility. OS is an austenitic file and instruments with austenitic crystal arrangement tend to be stiffer and harder [7]. It can be assumed that the type of thermomechanical process exploited by the manufacturers affects the behavior of the instrument within the root canal. Almeida et al. found no significant difference in apical transportation between the conventional superelastic K3 and the heat-treated R-Phase K3XF files when utilizing the same methodology to quantify canal transportation as used here [37]. However, Gagliardi et al. found superior results in terms of canal transportation after instrumentation with ProTaper Gold in comparison with the conventional ProTaper Universal [24]. The reason for these differing results could be that although K3XF is a heat-treated file, it is not martensitic at room temperature [38]. The advantages of high flexibility and controlled memory in martensitic instruments are only evident in files that have a high austenite finish temperature exceeding body temperature [7]. Consequently, the null hypothesis was rejected because of the significant differences in the canal curvature straightening and the apical transportation between the two types of files. Adequate knowledge of the root canal anatomy and the types of available instruments and equipment is essential for endodontic therapy success [39]. These results will help clinicians chose the appropriate file to tackle the different anatomical challenges during root canal treatment, such as severe curvatures. Clinically, endodontic instruments should maintain the original curvature of root canals, while transportation, as indicated by the excessive removal of dentin from the outer wall of curvature while preparing the apical third of root canals, should be avoided. Excessive transportation of the canal will cause other potential procedure misshapes (e.g., ledges, zipping, apical and strip perforations, canal blockage) that will eventually compromise the outcome of non-surgical root canal treatment [40]. The high flexibility of the novel thermomechanical treatment with the OC rotary file would retain the original root canal anatomy and help avoid procedural mishaps. A possible limitation of the presented study is the utilization of an ex vivo model. The study was done on extracted teeth and did not compensate for the intra-oral body temperature, with the experiment performed at room temperature. Body temperature can affect the phase transformation and clinical behavior of NiTi instruments [41].

## 5. Conclusions

Within the limitations of this study, the new OC thermomechanical treatment showed comparable increases in canal thickness, surface area, volume, and SMI score, with no significant differences from the conventional NiTi OS treatment. The instruments did not contact surfaces of the radicular dentin walls, with no significant difference in the percentages of untreated dentin surfaces. Even though both instruments were of the same design, the new OC thermomechanical treatment led to significantly less straightening of the canal curvature and transportation in the apical thirds of the canals compared to the conventional NiTi OS treatment.

## Figures and Tables

**Table 1:** Mean ± standard deviation of the absolute values and percentages of post-instrumentation changes in surface area; structure model index (SMI) score; canal thickness; volume of removed dentin; untreated canal surface; canal transportation in the coronal, middle, and apical thirds; and straightening of the canals.

Parameter	Group	Mean	*p-*Value
∆ Surface area, mm^2^	OS	2.61 ± 1.98	0.151 *
	OC	1.87 ± 1.65	
∆ SMI score	OS	0.80 ± 0.58	0.365 *
	OC	0.68 ± 0.58	
∆ Canal thickness, mm	OS	0.24 ± 0.09	0.458
	OC	0.22 ± 0.09	
The volume of removed dentin, mm^3^	OS	1.37 ± 0.72	0.093
	OC	1.02 ± 0.52	
Untreated canal surface, %	OS	54.52 ± 16.09	0.691
	OC	52.36 ± 17.83	
Straightening, %	OS	17.30 ± 11.86	0.039
	OC	10.77 ± 6.81	
Coronal transportation, µm	OS	103.57 ± 53.86	0.210
	OC	83.68 ± 44.39	
Middle transportation, µm	OS	66.37 ± 44.88	0.051
	OC	42.14 ± 28.96	
Apical transportation, µm	OS	55.11 ± 35.20	0.027 *
	OC	33.15 ± 22.47	

The third column shows the results of the independent sample *t-*test. * Refers to data that are not normally distributed according to Shapiro–Wilk test. Accordingly, Mann–Whitney *U* test was used for comparison.
